# Antioxidant and Anti-Inflammatory Effects of *Zingiber officinale roscoe* and *Allium subhirsutum*: In Silico, Biochemical and Histological Study

**DOI:** 10.3390/foods10061383

**Published:** 2021-06-15

**Authors:** Nourhene Zammel, Mohd Saeed, Nouha Bouali, Salem Elkahoui, Jahoor M. Alam, Tarek Rebai, Mohd A. Kausar, Mohd Adnan, Arif J. Siddiqui, Riadh Badraoui

**Affiliations:** 1 Laboratory of Histo-Embryology and Cytogenetics, Medicine Faculty of Sfax, University of Sfax, Sfax 3029, Tunisia; nourhene.zammel@gmail.com (N.Z.); tarek.rebai@fmsf.rnu.tn (T.R.); 2 Department of Biology, University of Ha’il, Ha’il 81451, Saudi Arabia; mo.saeed@uoh.edu.sa (M.S.); nouha_bmail@yahoo.fr (N.B.); s.elkahoui@uoh.edu.sa (S.E.); j.alam@uoh.edu.sa (J.M.A.); mo.adnan@uoh.edu.sa (M.A.); arifjamal13@gmail.com (A.J.S.); 3 Section of Biochemistry, University of Ha’il, Ha’il 81451, Saudi Arabia; ma.kausar@uoh.edu.sa; 4 Laboratory of Histology—Cytology, Medicine Faculty of Tunis, University of Tunis El Manar, La Rabta-Tunis 1007, Tunisia

**Keywords:** antioxidants, inflammation, oxidative stress, *Zingiber officinale roscoe*, *Allium subhirsutum*, inflammatory biomarkers, molecular docking, histopathology

## Abstract

In this study, the antioxidant and anti-inflammatory effects of *Zingiber officinale roscoe* and *Allium subhirsutum* aqueous extracts were examined in a carrageenan-induced acute inflammation model. Some markers of inflammation such as hematological parameters, fibrinogen and C-reactive protein were measured. Variables reflecting oxidative stress included thiobarbituric acid reactive substances (TBARS), advanced oxidation of protein products (AOPP), superoxide dismutase (SOD), catalase (CAT), glutathione peroxidase (GPx) and glutathione were determined in both inflamed foci and erythrocytes. The in silico molecular docking simulation showed that the main components of *Zingiber officinale roscoe* and *Allium subhirsutum* bound to toll-like receptor 6 (TLR6) with high affinities. Moreover, histological examinations of paw edema were carried out. Both *Zingiber officinale roscoe* and *Allium subhirsutum* ameliorated the induced inflammation and oxidative stress status as outlined by anti-edematous, antioxidant and anti-inflammatory activities. Our investigation lends pharmacological support to the medical uses of these spices in the management of inflammatory disorders and oxidative damage. The results of the in silico assay satisfactory explain the in vivo effects as compared with indomethacin.

## 1. Introduction

Paw edema induced by carrageenan (CAR), as described for the first time by Winter et al. [1], is a well-characterized experimental model of acute inflammation. The inflammatory process induced an increase in vascular permeability and cellular infiltration leading to paw edema, as a result of extravasation of fluid and accumulation of leukocytes at the inflamed tissue. In addition, the inflammatory response is associated with the production of reactive oxygen species (ROS), release of inflammatory agents and hematological changes. High production of ROS may contribute to cell damage by altering proteins, lipids and DNA/RNA [2,3,4]. It was well documented that ROS, themselves, can initiate the inflammatory process by enhancing the induction of pro-inflammatory cytokines and pro-inflammatory factors causing tissue injury [4,5,6]. Inflammatory cascade includes a wide range of receptors and pathways, including Toll-like receptors, which are commonly targeted during anti-inflammatory treatment. The acute inflammation is a very common disease. Enteritis, gastritis, bronchitis and other illnesses ending in “-itis” are dangerous related diseases in human based in acute inflammation.

Despite their important role in managing pain and inflammatory situations, the use of conventional anti-inflammatory drugs has discouraging profile of side effects [7]. This demonstrates the need for safe anti-inflammatory drugs based on bioactive substances such as flavonoids, which are found in most plants [8]. Plants are considered as a promising alternative to reduce oxidative damage and alleviate inflammation and several diseases thanks to their rich phytochemical contents such as polyphenols, flavonoids and tannins [8,9,10]. Previous studies have signaled the significant impact of those substances on the inflammatory process. Antioxidant, anti-inflammatory and anti-proliferative effects of plant extracts might involve different signaling pathways, especially TLR [11]. Likewise, it has been shown that the anti-inflammatory effect is commonly associated with inhibition of key inflammatory pathways: TLR6, IL-1, NFκB and TNFα signaling [12]. In fact, medicinal plants with such potency reduced the pro-inflammatory cytokines expression and inhibited TNFα and MAPK [13]. *Zingiber officinale roscoe* (ZO) and *Allium subhirsutum* (AS) are known as hot spices belonging to Zingiberaceae and garlic families respectively. Both species have tremendous contribution in health improvement and have been used in medical purposes [14,15,16]. Phytochemical studies showed that these slender perennial plants are rich in several bioactive phenolics, including non-volatile pungent compounds such as gingerols, paradols, shogaols, and zingerones [14,17,18]. Those constituents are the medicinally active substances which are responsible for the biological activities. Both plants exhibited potent health benefits including anti-oxidant and anticancer potential [15,19,20]. Hence, acute inflammation is a public health problem for which nutraceuticals can be beneficial. However, to what extent nutraceuticals, particularly ZO and AS extracts, could alleviate antioxidative injury associated acute inflammation is not well documented and might be of big interest to explore. Considering those potentialities, the present study aimed to investigate the antioxidant and the protective effects of both ZO and AS on acute inflammation using in silico, biochemical and histological assays. Accordingly, this study was carried out using a rat model of acute inflammation and targeting TLR 6 in the in silico molecular docking simulation.

## 2. Materials and Methods

### 2.1. Plant Preparation

Rhizomes of ginger, ZO and AS were procured from local markets in Sfax (Southern-Est of Tunisia) and Makkah (South of Saudi Arabia) respectively. The Voucher specimen numbers are BNM0000000963 for ZO and 7427 for AS. They are deposited in Beleu National Museum and EGE University’s Herbarium respectively. They were washed with distilled water, dried and then crushed into powder. A decoction for a minimum of 10 min was prepared from the same amount of crushed herb and water (W/V), until about 50% of the water is lost. ZO and AS samples were crushed using a marble stony mortar. During heating, the vessel was closed to prevent the loss of any essential constituents. The extract was then filtered using Whatman No.1 filter paper. The aqueous extracts were then further used to assess their antioxidant and anti-inflammatory effects in rats.

### 2.2. Animals

The experimental study was conducted on thirty Wistar rats aged from 8 to 10 weeks and weighting about 180–200 g. Animals were purchased from the Central Pharmacy of Tunisia (SIPHAT, Ben Arous, Tunisia). They were housed in metal cages at 23 ± 2 °C, 12/12 h light/dark cycle and were provided with water ad libitum and standard laboratory diet (SICO, Sfax, Tunisia).

### 2.3. Experimental Design and Drug Treatment

Animals were randomized into five groups (n = 6), and kept to acclimatize to the laboratory conditions for one week. This helps to keep stress levels low, which is important for the development of a good inflammatory response.

Group 1: Control; control rats, non-inflamed and received a subplantar injection of physiological saline (0.9%). This group served as negative control.

Group 2: CAR; inflamed rats by receiving a subplantar injection of 100 µL of CAR (1% of carrageenan suspension in (0.9% NaCl) [1]. This group served as positive and non-treated control. Carrageenan was purchased from Sigma Aldrich Company (Sigma chemical Company, St Louis, USA).

Group 3: CAR_ZO; rats treated with aqueous extract of ZO (100 mg/kg BW) for one week then received CAR injection the 8th day.

Group 4: CAR_AS; rats receiving aqueous extract of AS (100 mg/kg BW) for one week then injected with CAR the 8th day.

Group 5: CAR_IN: rats receiving intramuscular injection of indomethacin (IN) (100 mg/kg BW) after CAR injection (10 mg/kg of body weight). This group served as positive and treated control.

ZO and AS aqueous extracts have been given by gavage. Paw edema was induced by injection of CAR (1%) into the sub-plantar surface of the right paw of each rat. For the assessment of the anti-inflammatory activity, paw thickness was measured before and after the CAR injection with a digital caliper at different times (at 1, 2, 3, 4 and 5 h after CAR injection). The paw edema size was calculated by subtracting the initial paw thickness (before CAR injection) from the paw thickness measured at each hour (after CAR injection) using a Banggood Stainless Steel Electronic Vernier caliper, as previously reported [21,22].

Percentage of inhibition against edema formation was taken as an index of acute anti-inflammatory activity. Percentile of inhibition was calculated according to the following formulas:

**%** of inhibition = (Vc − Vt / Vc) * 100

where:

Vc = Volume of paw edema in control animals
Vt = Volume of paw edema in treated animals.

Five hours after CAR injection, animals were sacrificed and paws were removed by cutting at the tibio-tarsal level. Paws were then be used for further analyses. All animal experiments were approved by the local Ethical Committee (University of Sfax—Medicine Faculty, 12/ES/15—2018-20) and its guidelines which refer to the guide for the care and use of laboratory animals as adopted and promulgated by the United States National Institutes of Health.

### 2.4. Sample Preparation

Blood samples were collected by putting a funnel under the rat’s neck after decapitation following general anesthesia using 8% chloral hydrate (400 mg/100 g BW). Blood samples were collected in EDTA tubes and were immediately used for the determination of hematological parameters. Other tubes were used for serum separation by centrifugation. The serum samples were aliquoted and stored at –80 °C until analyzed. The sediment-containing erythrocytes were suspended in phosphate-buffered saline solution (0.9% NaCl in 0.01 M phosphate buffer, pH = 7.4) and centrifuged as reported by Sinha et al. [23]. Skin tissues, close to the inflammation foci, were homogenized (10%, W/V) with phosphate buffer saline (pH = 7.4) and centrifuged at 9000 rpm for 20 min. Both homogenized skin and erythrocytes were collected and used for oxidative stress testing.

### 2.5. Inflammatory Biomarkers

Both fibrinogen and C-reactive protein (CRP) were assessed in blood samples using high sensitivity enzyme immunoassay kits manufactured by MyBiosource, Inc. (San Diego, CA, USA). CRP is a specific and reliable marker of the inflammatory process. It increases in proportion to its intensity [24]. CRP was determined by the turbidimetric method using an automatic analyzer COBAS INTEGRA 400” C-Reactive. This parameter is expressed as mg/L.

Plasma fibrinogen level was determined according to Clauss’s method [25]. The principle of this test was to measure the conversion rate of fibrinogen into fibrin in diluted sample in the presence of excess of thrombin and records the clotting time. Fibrinogen level is inversely proportional to the clotting time. Tested fibrinogen samples are expressed in g/L of plasma.

### 2.6. Hematological Parameters

Blood was collected in EDTA-treated tubes for blood test including white blood cells count (WBC, 10^3^/µL), red blood cells count (RBC, 10^6^/µL) and platelets (PLT, 10^3^/mm^3^). These parameters were quantified using an automatic hematological assay analyzer (KX21 hemogram, CHU Habib Bourguiba, Sfax, Tunisia).

### 2.7. Oxidant Assays

The oxidative stress parameters were determined in inflamed area and erythrocytes and reported by the protein quantity (/mg of protein). Protein quantification in skin and erythrocytes was assessed according to the method described by Lowry et al. [26] using bovine serum albumin as standard.

Thiobarbituric acid reactive substances (TBARS) measurement was assessed following the method of Draper and Hadley [27] by examining the concentration of malondialdehyde (MDA). This detection is based on a reaction in which an MDA molecule reacts with two molecules of thiobarbituric acid resulting in the formation of a red chromogen at pH 2–3 and 95 °C. After mixture with two volumes of trichoroacetic acid (TCA, V/2V) and homogenation using an Ultra Turax homogenizer, the supernatant was allowed to react with an equal volume of TBA in water-bath for 15 min. The absorbance was measured at 532 nm and the amount of TBARS was expressed as nmol of MDA/mg of protein.

Advanced oxidation of protein products (AOPP) levels were determined in erythrocytes and skin according to Kayali’s method [28]. The absorbance of the reaction mixture was recorded at 340 nm, and the concentration of AOPP was calculated using the extinction coefficient of 261 cm/mM, and the results were expressed as nmoles of AOPP/mg of protein.

### 2.8. Antioxidant Assays

Superoxide dismutase (SOD) activity was estimated in supernatant fractions based on the method developed by Beauchamp and Fridovich [29] based on the ability to inhibit the photoreduction of nitroblue tetrazolium (NBT) at 25 °C. One unit of SOD activity was determined as the amount of enzyme that inhibited 50% of the NBT reduction. The reaction product was measured at 560 nm, and the values were expressed as Units/mg of protein.

Measurement of catalase activity (CAT) was spectrophotometrically analyzed at 240 nm according to Aebi’s method [30] by measuring the decrease of H_2_O_2_ at 240 nm for 60 s. One unit of catalase activity catalyzed the degradation of 1 µmol of H_2_O_2_ per min. The enzyme activity was expressed as μmoles H_2_O_2_ consumed/min/mg of protein.

Glutathione peroxidase (GPx) activity was analyzed according to the method of Flohé and Gunzler [31]. The principle of this method is based on the conversion of the oxidized reduced glutathione to the reduced form with a concomitant oxidation of NADPH–NADP+. The enzyme activity was expressed as µmoles of GSH oxidized/min/mg of protein.

Glutathione (GSH) level was determined according to Ellman [32] modified by Jollow et al. [33]. The method was based on the changes in absorbance resulting from the conversation of NADPH into NADP. The GSH content was expressed as μg/mg of protein.

### 2.9. Histological Examination

Biopsies of inflamed site were collected for the histopathological examination purposes. Representative paw tissue from each group was fixed in 10% neutral buffered formalin solution, embedded in paraffin, cut into 5 µm thick sections and stained with hematoxylin-eosin (H&E). The slides were examined and photographed with a Canon camera (16 MG) connected to an optical microscope (Olympus Inc., Tokyo, Japan).

### 2.10. In Silico Molecular Docking Assay

The tridimensional structure of TIR domain of Toll-like receptor 6 (TLR6, particularly 4OM7, DOI:10.2210/pdb4OM7/pdb) and the main components of both ZO and AS were obtained from RCSB and PubChem websites respectively. A blind binding based on the CHARMm force field was used in docking. The ligands were assessed for their ability to the crystal structure of TRL6 (4OM7) after removing water molecules and adding polar hydrogens and Kollman charges as previously reported [10,34]. As a TLR, the 4OM7 play a critical role in the first line of defense (innate immune responses) including inflammation by transmembrane interactions in human [35].

### 2.11. Statistical Analysis

The obtained data were expressed as mean and standard error of the mean (mean ± SEM). The one-way analysis of variance (ANOVA) and the Tukey post hoc test were performed on the data for intergroup comparisons using SPSS version 18. Significant differences were considered whenever *p* < 0.05.

## 3. Results

### 3.1. Anti-Inflammatory Activity

**Figure 1:**
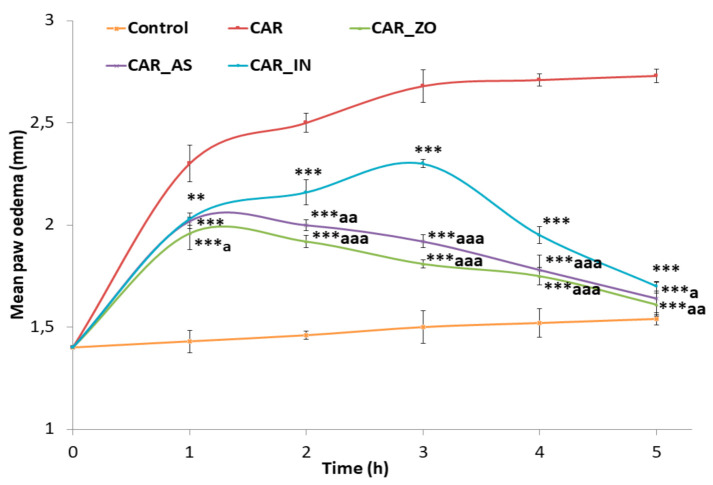
Changes in edema size after CAR injection in experimental animals: Control: non-treated rats, received injection with isotonic saline solution (NaCl, 0.9%); CAR: inflamed rats by Carrageenan (1%); CAR_ZO, CAR_AS and CAR_IN: rats inflamed by CAR and treated with ZO, AS or indomethacin respectively. *, **, *** for *p* < 0.5, 0.01 and 0.001 vs. CAR and ^a^, ^aa^, ^aaa^ for *p* < 0.5, 0.01 and 0.001 vs. CAR_IN.

**Figure 2:**
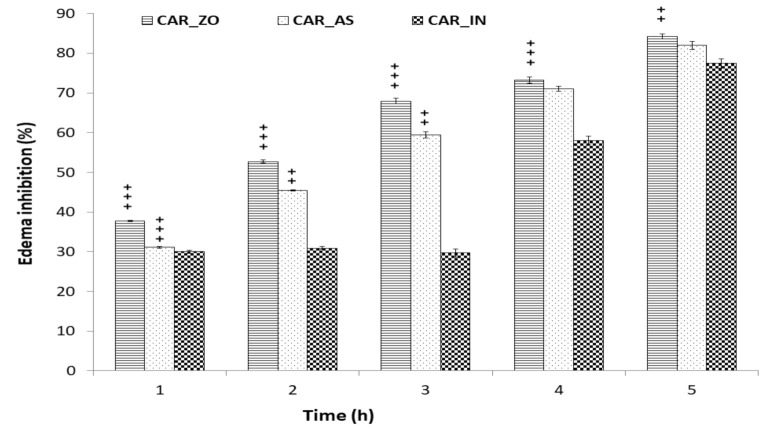
Percentage of inhibition of edema produced by ZO, AS and IN alone at various time intervals of CAR-induced rat paw edema: CAR_ZO: rats pre-treated with ZO for 7 days before CAR injection; CAR_AS: rats treated with AS after CAR injection and CAR_IN: rats treated with IN after CAR injection. Values are expressed as mean ± SEM. Values are expressed as mean ± SEM. Symbols (^+^) exhibit significant statistical differences between the groups. ^+^ *p* < 0.05; ^++^ *p* < 0.01 and ^+++^ *p* < 0.001 versus CAR_IN group.

Changes of edema size are presented in Figure 1. The edema size and the percentage of inflammation increased rapidly to reach a maximum around the 3rd hour after CAR injection in the inflamed groups. However, these increases were less important in groups treated with ZO, AS or with IN. Rats treated with either ZO or AS for one week before CAR injection showed significant decrease in paw edema thickness following CAR injection (Figure 1). At the 5th hour of treatment, the decrease in paw edema size and the percentage of inflammation in all treated groups was highly significant when compared to CAR group. As illustrated in Figure 2, treatment with ZO or AS showed the largest effect in reducing paw edema thickness as compared with the reduction produced by the reference drug (indomethacin).

The suppression of the edema was statistically significant from the 4th hour in the group treated with indomethacin (CAR_IN). The inhibition was 30% (at the 1st hour) to reach 58% (at the 4th hour) and 71% (at the 5th hour). However, treatment with ZO or AS showed anti-inflammatory effects from the 2nd hour after CAR injection. Nevertheless, the most significant anti-inflammatory response occurred at the 5th hour by more than 80% of edema inhibition (Figure 2).

### 3.2. Inflammatory Biomarkers

At the 5th hour following the CAR injection, blood samples were analyzed for the inflammatory biomarkers CRP and fibrinogen. As depicted in Table 1, injection of CAR resulted in significant increased (*p* < 0.001) CRP and fibrinogen in the inflamed group (CAR) when compared to the control group. Furthermore, treatment with ZO or AS have significantly prevented CAR-induced elevation in those two markers. The effect of both ZO and AS was comparable to the reference used compound, indomethacin.

### 3.3. Hematological Parameters

As shown in Table 2, subplantar injection of CAR produced significant changes in hematological parameters (WBC, RBC and PLT). In comparison with the control group, highly significant rises in WBC and PLT were observed. However, RBC level was found reduced in inflamed rats (CAR). All treatments administrated to inflamed rats restored those parameters to near normalcy. The anti-inflammatory effect of with ZO was highly significant.

### 3.4. Oxidative Stress Assessment

Departing from Table 3, we infer that injection of CAR increased both TBARS and AOPP levels in both skin and erythrocytes of inflamed group when compared to healthy rats. The administration of ZO, AS and IN alone significantly decreased the level of those markers. Nevertheless, the protection conferred by the ZO and AS alone was more effective (*p* < 0.001) than that conferred by IN.

In order to explore the effects of anti-oxidant defenses on the inflammation process in skin and erythrocytes, the anti-oxidant activities of SOD, CAT and GPx were carried out. GSH level was also determined. Results presented in Table 3 showed that SOD, CAT and GPx activities were significantly lower in CAR group when compared to control rats. GSH content in CAR injected group was also reduced. In contrast to CAR injected paws, treatment with ZO, AS or IN have significantly restored those changes to near normalcy. However, pre-treatments with ZO or AS extract corrected the oxidative damage to a greater degree (*p* < 0.001) than treatment with IN (Table 3).

### 3.5. Molecular Docking Findings

**Figure 3:**
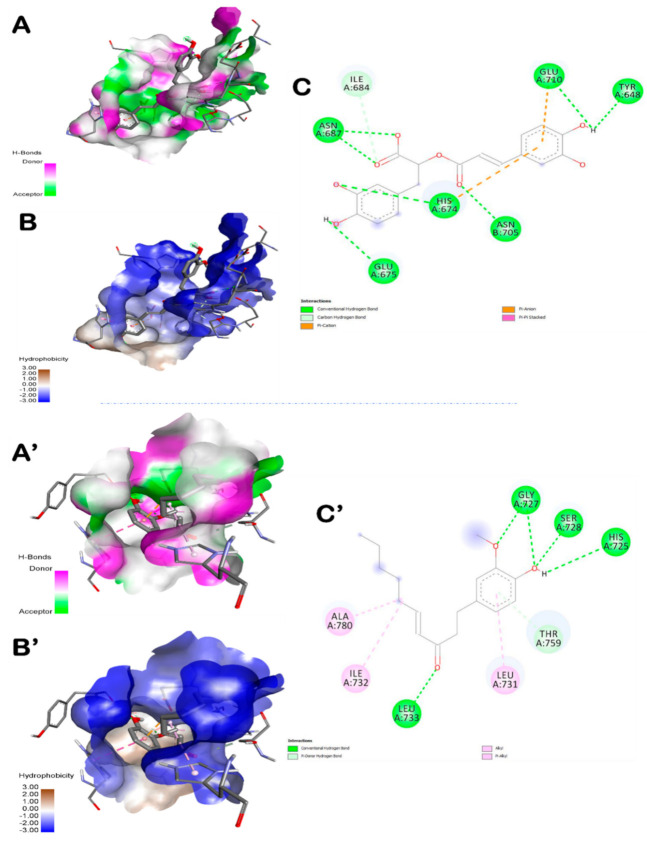
Representation of the 3D structure of ZO compounds (6-shogaol (**A**–**C**) and rosmarinic acid (**A’**–**C’**)), with the highest docking scores, bounded to the pocket region of TLR6. Micrographs of the pocket region with hydrogen bond (**A**,**A’**) and hydrophobicity illustrations (**B**,**B’**) and the corresponding 2D diagram of interactions (**C**,**C’**).

Table 4 exhibits the binding affinities, the number of conventional H-bonds and the distance to the closest interacting residue. The different ZO assessed compounds exhibited various binding affinities but all of them showed negative binding energy ranging from −5.4 to −10.8 kcal/mol. The highest binding score was found with amentoflavone (−10.8 Kcal/mol) followed by rosmarinic acid (−8.6 Kcal/mol). The latter exhibited seven hydrogen bonds. It was bonded to Asn687 (2x), His674, Asn705, Glu675, Glu710 and Tyr648 (Figure 3). AS compounds exhibited various binding affinities but all of them showed negative binding energy ranging from −5.2 to −7.7 kcal/mol.

**Figure 4:**
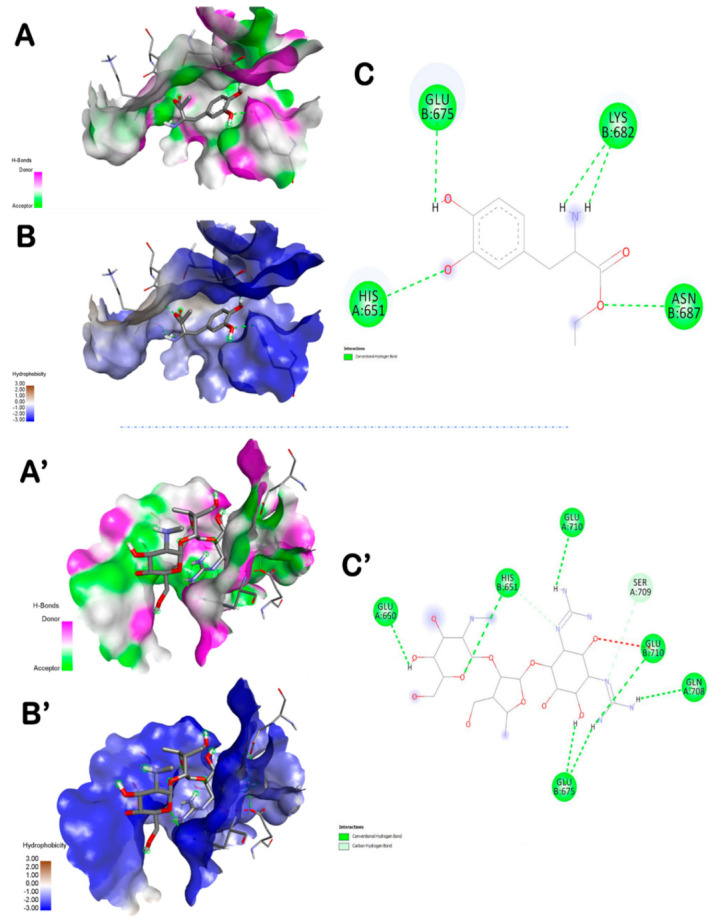
Representation of the 3D structure of AS compounds (L-4-hydroxy-3-methoxy-amethylphenylalanine (**A**–**C**) and dihydrodeoxystreptomycin (**A’**–**C’**)), with the highest docking scores, bounded to the pocket region of TLR6. Micrographs of the pocket region with hydrogen bond (**A**,**A’**) and hydrophobicity illustrations (**B**,**B’**) and the corresponding 2D diagram of interactions (**C**,**C’**).

**Figure 5:**
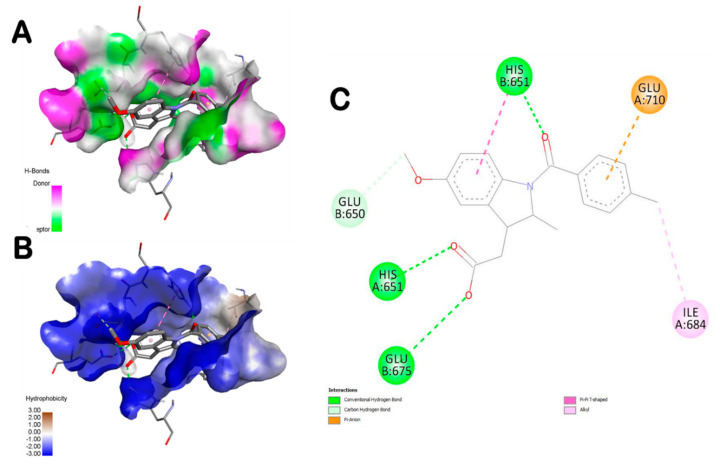
Representation of the 3D structure of indomethacin bounded to the pocket region of TLR6. Micrographs of the pocket region with hydrogen bond (**A**) and hydrophobicity illustrations (**B**) and the corresponding 2D diagram of interactions (**C**).

The highest number of hydrogen bonds was established with dihydrodeoxystrptomycin. It exhibited one hydrogen bond with each of Glu650, His651, Glu710, Ser709, Glu675, Gln708 (Figure 4). In this study, the in silico molecular docking findings were compared with indomethacin as for the in vivo assay. Table 5 and Figure 5 showed that the interactions of some ZO and AS compounds were much more competitive than indomethacin, in term of binding affinity and conventional hydrogen-bonds.

### 3.6. Histological Findings

**Figure 6:**
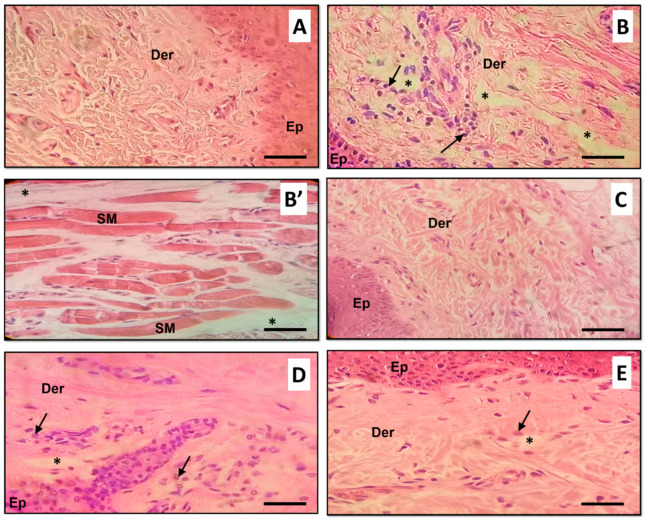
Histopathological examination of specimens from rat paw tissues of all studied groups. (**A**) Control rat treated with physiological saline (NaCl, 0.9%) showing no histopathological changes; (**B**) CAR rat received Carrageenan (CAR, 1%) showing marked inflammatory reaction in the dermis associated intermuscular infiltration with massive number of neutrophils and marked dermis and intermuscular edema (**B’**); (**C**,**D**) CAR_ZO and CAR_AS rats treated with ZO or AS respectively for 7 days before CAR injection showing very weak inflammatory reaction once compared with CAR slide; (**E**) CAR_IN rat treated with indomethacin after CAR injection showing moderate inflammatory reaction. Ep: epidermis, Der: dermis, SM: skeletal muscle cells, Arrows: inflammatory cells infiltration, Asterisks: edema. Scale bar = 75 µm.

The histopathological severity of the inflammatory response in paw tissue specimens from different groups was scored according to the histopathological alterations. Normal rats revealed normal histological structure of both epidermis and dermis (Figure 6A). Meanwhile, CAR injection resulted in acute edematasis especially in the dermis and between the muscular fibers associated with diffused leucocytes, particularly PMN (Figure 6B,B’). Examined sections from rats treated with ZO or AS (Figure 6C,D respectively) for one week before CAR injection showed very weak inflammatory reaction with very few focal inflammatory cells infiltration and edema. Treatments with IN after CAR injection reduced the histological changes induced by CAR challenge. Sections revealed moderate inflammatory response associating slight edema and neutrophils infiltration (Figure 6E).

## 4. Discussion

Carrageenan-induced edema in the rat paw is a widely used model to investigate the physiopathology of acute local inflammation, and to test the potential anti-inflammatory effect of new molecules. The present investigation outlines the anti-inflammatory and antioxidant activities of ZO and AS aqueous extracts, as nutraceuticals, against experimental CAR-induced acute inflammation using biochemical, histological and in silico assays. The results of the different approaches paralleled each other and confirmed the beneficial effects of ZO and AS extracts on this public health problem. Lambda (λ)-carrageenan is used to induce acute inflammatory reactions accompanied by hyperalgesia [36]. Localized injections of CAR caused infiltration of neutrophils, plasma extravasation of proteins at the site of injection and increase in vascular permeability associated with tissue edema [36,37]. The arachidonic acid cascade is highly activated during inflammation and it is mediated by cyclooxygenase and 5-lipoxygenase enzymes [38]. Although drugs with anti-inflammatory action are known to down regulate inflammation, the role of anti-oxidant mechanism pathway in mediating the action of such agents has not yet been elucidated.

The inflammatory response to CAR consisted of two phases [1,39]. The first phase (1–3 h after administration) is mediated by histamines, serotonins and kinins. The prostaglandins (PGs) seem to be mediators of the second phase (3–5 h after administration) [40]. In the current study, ZO and AS extracts seemed to be able to inhibit CAR-induced paw edema in the first and second phases (Figure 1 and Figure 2). Hence, the anti-edematous activity of both ZO and AS might be important during the prostaglandin phase but also effective during the first phase of inflammation. These results suggested that ZO and AS could inhibit rat paw edema by acting as a serotonin antagonist and also by inhibiting PGs production. Our findings gain support by previous reports indicating that ginger and garlic constituents may suppress the inflammation process by inhibiting arachidonic acid metabolism. Gingerols, as major components of ZO, have been reported to cause suppression of both cyclooxygenase and lipooxygenase metabolites and arachidonic acid [41]. Experimental studies reported that phytochemicals may inhibit PGs synthesis in vitro [42]. Further studies on the effects of ZO and AS extracts on pro-inflammatory mediators such as histamines, serotonins and kinins would support and confirm the findings of the current study.

Commonly, the anti-inflammatory process involved reduction in nuclear factor-κΒ (NF-κB) and interleukin (IL)-1b levels [43]. In this context, ZO and AS may decrease this factor, particularly TNF-α production. The effect may be explained either by blocking activation of this pro-inflammatory mediator and its transcriptional regulator [44], and/or inhibits its production by macrophages [45]. The mechanism underlying the inhibition of the edema, in this experimental study, could possibly be based upon an inhibition of both 1st and 2nd phase of inflammation due to their phyto-constituents, particularly flavonoids [8]. Their phytochemical profile, as previously reported, could inhibit the production/release of inflammatory mediators like serotonins, histamines, and PGs.

To better understand the mechanism of anti-inflammatory activity of ZO and AS aqueous extracts, their effects on CRP level was further studied. Inflammatory reactions trigger acute phase of proteins synthesis, such as CRP. This protein is produced essentially in the liver, as it can also be produced by local inflammatory cells in the area of damaged tissues [46]. Changes in acute phase proteins reflect the presence and the intensity of inflammation. Our findings regarding elevated levels of CRP (Table 1) go in harmony with Vazquez et al. [47], who suggested that the increased level of CRP was implicated into the expression of tissue factor and IL-6 in both paw and lung of CAR rat model. Treatment with plant extracts decreased the CRP levels, similarly to IN drug. However, rats treated with ZO or AS for one week exhibited lower CRP level. This indicates that tested spices have potential inhibitory effects on acute inflammation.

Plasma fibrinogen levels are increased under inflammatory situations [48]. According to our data, high fibrinogen levels were evident 5 h after CAR injection. Increased fibrinogen concentrations have been associated with an elevated risk of thrombotic diseases [49]. It could be deduced, as previously reported, that thrombin and fibrin could stimulate mononuclear cells and endothelial cells to synthesize interleukin 6 (IL-6) and interleukin 8 (IL-8) [50]. High fibrinogen levels have been associated with increased platelet aggregation, an important factor for the genesis of vascular lesions [51]. In this context, platelet aggregation and fibrin deposition were occurred [52]. These inflammatory biomarkers, which had been increased following the inflammation response, were lowered back to control levels through the use of ZO, AS or indomethacin (Table 1). The alleviative effect is certainly related to the phytochemical profiling of the plant extracts as previously reported and shown in Table 4.

In the present study, RBCs count was decreased in inflamed rats (Table 2). It is known that ROS influence RBCs deformability and degradation in inflammation [53]. ZO, AS and IN increased the RBCs count which was reduced through inflammation induction. CAR-induced paw edema was reported to be associated with changes in others blood parameters leading to a hemostatic imbalance. Based on our observations, we demonstrated an increase in WBCs and platelet aggregation 5 h following inflammation induction (Table 2). Our results go in good with Cicala et al. who found that the WBCs count and platelet reactivity were increased in inflamed rats. This increase occurred at the site of inflammation was due to the release of inflammatory cytokines, which increases the recruitment of neutrophil counts. It has been proven that the platelets form aggregates with leukocytes and interact with neutrophils, monocytes, and lymphocytes [9]. The reduced count of WBCs and platelets following treatment with ZO or AS was significantly higher than that observed in IN group after CAR injection. These findings suggested that phytoconstituents of both plants are able to reduce inflammation through decreasing leukocyte migration to the paw injury sites and inhibiting neutrophils and granulocytes infiltration.

The excessive production of ROS at the site of inflammation is proposed to be a major cause of the cell and tissue damage including cancer and inflammatory diseases [3,4,6,54,55,56]. Pro-inflammatory cytokines have been implicated in the release of ROS. AOPP are markers of protein oxidation as a result of the action of free radicals generated by activated neutrophils and involved in inflammation [57,58]. In the current work, CAR injection in rat paw induced ROS generation, as indicated by elevation of both TBARS and AOPP levels, and overwhelmed SOD, CAT, GPx and GSH (Table 3). Increased ROS formation in the extracellular space was seen in the inflammatory state in which low concentrations of SOD, CAT and GPx increased the susceptibility of extracellular components to ROS injury and may stimulate chemotaxis for other inflammatory cells [57,59]. The results of this study demonstrated that both treatment with ZO, AS or IN reduced significantly TBARS and AOPP formation in paw and erythrocytes. We established that ZO and AS increased SOD activity, which was inhibited by CAR in rat paw tissues and erythrocytes. CAT reacts with H_2_O_2_ to form water and molecular oxygen by donating hydrogen. In this study, we outlined that ZO and AS enhanced CAT activity which was reduced in CAR group. The anti-inflammatory effects of the ZO can be supported by GSH level and GPx activity. Furthermore, ZO and AS alleviated the decrease in GPx activity and restored the depleted GSH content in the assessed tissues. Both ginger and allium are ranked as plants with highest antioxidant properties [14,18,60,61] by inhibiting ROS generation, as proven in our results by restoring the redox state, offers another explanation of the anti-inflammatory activity of these spices.

Phytochemical studies showed that ZO is rich in a large number of compounds including gingerols, shogaols, hexadecanoic acid, and tuberonic acid [8]. These substances have been reported to display anti-inflammatory activity [62]. Recently, 6-gingerol, 8-gingerol and 10-gingerol, active ingredients in ginger, were reported as diphenyl-1-picrylhydrazyl (DPPH), super oxide and hydroxyl radical scavenging agents [63]. Other compounds of ginger rhizome including paradols and shogaols revealed strong inhibition of COX-2 enzyme which is involved in cancer and inflammatory responses [56,64].

In an attempt to rationalize the anti-inflammatory effect of ZO and AS, a molecular docking assay was carried out between the main bioactive compounds encountered in ZO and AS extracts (as reported by previous studies, including those realized by our team, using HPLC and HR-LCMS [14,17,41]) and the human TLR6. Molecular docking simulation can be efficient to understand and predict the structure-activity relationship between the ZO and AS bioactive compounds and some receptors involved in inflammation development such as TLR6. Hence, the main ZO and AS compounds were simulated with the TLR6, the number of established hydrogen bonds and also the receptor interacting residues. In this study, indomethacin was used as a reference drug in the in vivo approach but also in the in silico and docking simulation. The results showed that the main ZO or AS compounds interacted with TLR by establishing more hydrogen bonds than indomethacin and were much more deeply embedded in the receptor pocket (Table 4). The best poses were stabilized by seven hydrogen bonds for both ZO and AS compounds (Figure 3, Figure 4 and Figure 5). These results confirmed previous reported data about the effects of some phytochemical compounds belonging to ginger and garlic as assessed by in silico and molecular docking [14,65,66]. Indomethacin established three hydrogen bonds only together with lower interaction score. Hence, indomethacin seemed non-competitive with 6-shogaol, rosmarinic acid, L-4-hydroxy-3-methoxy-amethylphenylalanine and dihydrodeoxystreptomycin. These compounds were further detailed and compared to indomethacin interactions (Table 5). Apart from rosmarinic acid, which established bonds with different residues once compared with indomethacin, all the other selected compounds interacted with 2 to 4 key same residues of the pocket region as compared with the reference compound (IN). Moreover, Glu675 and His651 were the most involved TLR6 residues. This may justify their important role in the TLR6 binding and also inhibition during the inflammatory process [67]. The 3D illustration and the 2D diagram of interactions exhibit that the selected compounds bound to TLR6 through a network of electrostatic, hydrophobic and hydrogen bonds. Dihydrodeoxystreptomycin showed the highest number of hydrogen bonds associated the highest number of closest interacting residues, which suggest that this compound might have the strongest anti-inflammatory effect. Table 5 exhibits that AS selected compounds (L-4-hydroxy-3-methoxy-amethylphenylalanine and dihydrodeoxystreptomycin) occupied almost the same region (His651 and Glu675). This may suggest that synergistic effect is possible. Accordingly, It was supported that the use of the whole plant extract might be much better [8,10].

Amentoflavone exhibited the highest binding score, however, rosmarinic acid filled into the active site with seven conventional hydrogen bonds associated 1 carbon hydrogen bond (Ile684), 2 Pi-anion/cation (Glu710, His674) bond and 1 Pi-Pi stacked bond (His674) with different residues of the active site. These findings confirm previous studies on TRL6 which was interpreted to be involved in the development of immunotherapeutic agents via docking assays of N-acetylated Pam_2_Cys [68]. Furthermore, all the assessed compounds showed negative binding energy, which could explain the ZO anti-inflammatory effect as outlined by the biochemical and histological assays. In fact, paw histological micrographs from CAR group showed focal inflammatory cell infiltration, essentially neutrophils, which lead to perturbation of the inflammatory system. This fact was documented in several studies carried out on the same animal model [61]. Treatment with ZO or AS considerably decreased inflammatory cell infiltration and reduces edema as did IN (Figure 6). In fact, the treatment for one week before inducing inflammation alleviated these histopathological features.

## 5. Conclusions

In summary, our study provides new evidences that a local CAR injection induces a systemic response, mainly characterized by changes in blood parameters, increased levels of acute phase proteins, CRP and fibrinogen, increased platelet reactivity, edema and leukocyte infiltration into injured site. The results of this animal study reveal that ZO and AS extracts possessed higher anti-inflammatory and antioxidant values. Pre-treatment with ZO or AS for one week has shown better inhibitory effect on various inflammatory biomarkers than the reference drug IN. The findings suggested that the antioxidant associated anti-inflammatory potentials of ZO and AS might involve TLR6 pathways and the inhibition of the release of inflammatory mediators, such as serotonin, histamine, and PGs as assessed by in silico molecular docking. These potentialities lend pharmacological support to medical uses of these spices in the management of inflammatory disorders and oxidative damage.

## Figures and Tables

**Table 1:** Inflammatory biomarkers in the serum of the different experimental rats: control rats, inflamed rats with CAR (CAR), inflamed rats pre-treated with ZO (CAR_ZO), inflamed rats treated with AS (CAR_AS) and inflamed rats treated with IN (CAR_IN).

Parameters	Control	CAR	CAR_ZO	CAR_AS	CAR_IN
CRP (mg/L)	0.79 ± 0.012	1.043 ± 0.015 ***	0.813 ± 0.015 ^###^	0.84 ± 0.02 ^###^	0.857 ± 0.009 *^###^
Fibrinogen (g/L)	1.743 ± 0.024	2.047 ± 0.009 ***	1.807 ± 0.003 ^###^	1.86 ± 0.2 ^###^	1.863 ± 0.029 *^##^

Values are expressed as mean ± SEM. Symbols (*, ^#^ and ^+^) exhibit significant statistical differences between the groups. * *p* < 0.05; ** *p* < 0.01 and *** *p* < 0.001 versus control group, ^#^ *p* < 0.05; ^##^ *p* < 0.01 and ^###^ *p* < 0.001 versus CAR group.

**Table 2:** Hematological parameters in the different experimental rats: control rats, inflamed rats with CAR (CAR), inflamed rats and treated with ZO (CAR_ZO), inflamed rats treated with AS (CAR_AS) and inflamed rats treated with indomethacin (CAR_IN).

Parameters	Control	CAR	CAR_ZO	CAR_AS	CAR_IN
WBC (10^3^/µL)	12.95 ± 0.386	19.1 ± 0.785 ***	13.27 ± 0.549 ^###^	14.33 ± 0.94 *^###^	14.175 ± 0.437 ^##^
RBC (10^6^/µL)	8.1 ± 0.071	7.43 ± 0.025 ***	7.958 ± 0.040 ^###^	8.22 ± 0.41 ^###^	7.873 ± 0.071 *^##^
PLT (10^3^/mm^3^)	482.5 ± 7.773	924 ± 5.292 ***	505.5 ± 7.171 ^###++^	569 ± 46.72 *^###^	581.5 ± 16.102 ***^###^

Values are expressed as mean ± SEM. Symbols (*, ^#^ and ^+^) exhibit significant statistical differences between the groups. * *p* < 0.05; ** *p* < 0.01 and *** *p* < 0.001 versus control group, ^#^ *p* < 0.05; ^##^ *p* < 0.01 and ^###^ *p* < 0.001 versus CAR group, ^+^ *p* < 0.05; ^++^ *p*<0.01 and ^+++^*p* < 0.001 versus CAR_IN group.

**Table 3:** Evaluation of oxidative and antioxidant statutes in skin tissue and erythrocytes: control rats, inflamed rats with CAR (CAR), inflamed rats and treated with ZO (CAR_ZO), inflamed rats treated with AS (CAR_AS) and inflamed rats treated with indomethacin (CAR_IN).

	Control	CAR	CAR_ZO	CAR_AS	CAR_IN
Skin Tissue					
TBARS (nmoles /mg protein)	0.404 ± 0.02	1.076 ± 0.047 ***	0.455 ± 0.019 ^###+^	0.467 ± 0.051 ^###+^	0.604 ± 0.03 **^###^
AOPP (nmoles/mg protein)	0.613 ± 0.025	1.05 ± 0.037 ***	0.624 ± 0.016 ^###^	0.633 ± 0.074 ^###^	0.754 ± 0.019 **^###^
SOD (Units/mg protein)	4.917 ± 0.373	1.934 ± 0.179 ***	4.321 ± 0.148 ^###++^	3.692 ± 0.566 ^###^	3.724 ± 0.062 *^##^
CAT (µmoles/min/mg protein)	7.675 ± 0.407	2.697 ± 0.163 ***	7.237 ± 0.196 ^###++^	6.551 ± 0.892 *^###+^	6.232 ± 0.132 *^###^
GPx (µmoles/min/mg protein)	1.292 ± 0.045	0.591 ± 0.036 ***	1.185 ± 0.059 ^###+^	0.966 ± 0.093 *^###^	0.944 ± 0.014 ***^###^
GSH (µg GSH/mg protein)	1.181 ± 0.021	0.673 ± 0.021 ***	1.251 ± 0.039 ^###++^	0.846 ± 0.063 *^###+^	0.993 ± 0.028 **^###^
**Erythrocytes**					
TBARS (nmoles/mg protein)	1.881 ± 0.066	3.044 ± 0.12 ***	1.9 ±0.036 ^###++^	2.07 ± 0.102^###++^	2.254 ± 0.105 *^##^
AOPP (nmoles/mg protein)	0.938 ± 0.056	1.738 ± 0.07 ***	0.949 ± 0.036 ^###++^	1.017 ± 0.053^###++^	1.176 ± 0.069 *^##^
SOD (Units/mg protein)	21.111 ± 0.832	14.717 ± 0.384 ***	20.158 ± 0.62 ^###^	19.461 ± 0.663^###^	18.506 ± 0.285 *^###^
CAT (µmoles/min/mg protein)	16.404 ± 0.551	9.402 ± 0.522 ***	17.154 ± 0.256 ^###+++^	15.086 ± 0.774 *^###^	14.081 ± 0.495 *^##^
GPx (µmoles/min/mg protein)	3.373 ± 0.07	1.065 ± 0.61 ***	3.727 ± 0.138 ^###+++^	3.022 ± 0.427 ^###++^	2.34 ± 0.181 **^###^
GSH (µg/mg protein)	2.296 ± 0.04	1.446 ± 0.038 ***	2.443 ± 0.12 ^###++^	1.983 ± 0.056 *^###^	1.943 ± 0.057 **^###^

Values are expressed as mean ± SEM. Symbols (*, ^#^ and +) exhibit significant statistical differences between the groups. * *p* < 0.05; ** *p* < 0.01 and *** *p* < 0.001 versus control group, ^#^ *p* < 0.05; ^##^ *p* < 0.01 and ^###^ *p* < 0.001 versus CAR group, ^+^ *p* < 0.05; ^++^ *p* < 0.01 and ^+++^ *p* < 0.001 versus CAR_IN group.

**Table 4:** Ligands and human toll-like receptor 6 (TLR6) interactions: binding affinity, number of conventional hydrogen bonds and distance to the closest interacting residue.

Plant/Compound	Binding Affinity (kcal/mol)	Intermolecular INTERACTIONS	
		Conventional Hydrogen Bonds	Closest Interacting Residue (Distance, Å)
***Zingiber officinale rosea* L.**			
6-Gingerol	−7.1	4	His651 (2.321)
8-Gingerol	−6.6	3	Glu710 (2.208)
10-Gingerol	−6.0	3	Tyr648 (2.128)
6-Shogaol	−7.2	5	Ser728 (2.105)
Caffeic Acid	−6.1	3	Lys769 (1.980)
Rosmarinic Acid	−8.6	7	His674 (2.061)
Syringic Acid	−5.4	5	Ser728 (2.071)
Amentoflavone	−10.8	5	His674 (2.044)
Ferulic Acid	−6.3	3	Gln757 (2.363)
***Allium subhirsutum* L.**			
Methyl N-(amethylbutyryl) glycine	−5.3	3	His674 (2.187)
Bis (2-hydroxypropyl) amine	−4.4	3	Tyr648 (2.279)
Cepharanthine	−6.7	1	Asn705 (1.829)
(22S)-1alpha,22,25-trihydroxy-26,27-dimethyl-23,23,24,24-tetradehydro-24ahomovitamin D3/(22S)-1al	−7.7	2	Glu650 (2.312)
L-4-Hydroxy-3-methoxy-amethylphenylalanine	−6.1	5	Glu675 (2.204)
N-(2-fluro-ethyl) arachidonoyl amine	−7.1	1	His674 (2.731)
Dihydrodeoxystreptomycin	−6.2	7	Glu650 (2.069)
6 alpha-hydroxy castasterone	−6.7	4	Glu710 (2.045)
C16 Sphinganine	−5.2	3	Glu650 (2.172)
3beta,7alpha,12alpha-Trihydroxy-5alpha-cholestan-26-oic acid	−6.6	4	Glu710 (1.930)
4-Oxomytiloxanthin	−7.4	3	Asn705 (2.298)
Sebacic acid	−5.3	5	Tyr714 (1.866)
Tuberonic acid	−6.0	5	His713 (2.200)
6-Deoxo castasterone	−6.6	4	Gly681 (2.156)
2,2-difluoro-hexadecanoic acid	−5.3	5	Tyr714 (2.009)
**Indomethacin**	−6.2	3	His651 (1.969)

**Table 5:** Interacting residues of the main ZO and AS docked compounds into the human TLR6. Indomethacin was used a reference compound.

Compound Name	Interacting Residues in the Pocket Region of TLR6
6-Shogaol	*Ile684*, **Asn687**, ***Glu675***, **His674**, **Asn705**, ***Glu710***, **Tyr648**
Rosmarinic acid	Ala780, Ile732, **Leu733**, Leu731, Thr759, **His725**, **Ser728**, **Gly727**
L-4-Hydroxy-3-methoxy-amethylphenylalanine	***His651***, ***Glu675***, **Lys682**, **Asn687**
Dihydrodeoxystreptomycin	***Glu650***, ***His651***, ***Glu710***, Ser709, ***Glu675***, **Gln708**
Indomethacin (Reference)	**Glu675**, **His651**, Glu650, Glu710, Ile684
Bold amino acids: interacting with the correspondent compound via conventional H-bonds. Italics’ amino acids: same interacting residues as for the reference compound.	
